# Supplementary material for: Active Commuting to University Is Positively Associated with Physical Activity and Perceived Fitness
Source: Healthcare (Basel). 2022 May 26;10(6):990. doi: 10.3390/healthcare10060990 (PMC9222817; doi:10.3390/healthcare10060990)
Supplement: Supplementary file 1 [file healthcare-10-00990-s001.zip › healthcare-1672154-supplementary.pdf]

Table S1. Associations between mode of commuting with physical activity, sedentarism, and fitness of participants by gender.

	**Mode of Commuting to University ***			
	**Men**		**Women**	
	**Active**	**Public**	**Active**	**Public**
	**OR (95% CI)**	**OR (95% CI)**	**OR (95% CI)**	**OR (95% CI)**
**Physical Activity and Sedentarism**				
PA Levels				
Light	Ref.	Ref.	Ref.	Ref.
Moderate	**3.206**	**2.405**	1.278	0.973
	**(1.33, 7.69)**	**(1.04, 5.56)**	(0.63, 2.58)	(0.52, 1.73)
Vigorous	**2.674**	**2.583**	**2.729**	2.020
	**(1.18, 6.04)**	**(1.20, 5.53)**	**(1.10, 6.74)**	(0.90, 4.51)
MVPA recommendations				
Not meeting	Ref.	Ref.	Ref.	Ref.
Meeting	**2.298**	**2.071**	**2.073**	1.580
	**(1.16, 4.54)**	**(1.08, 3,96)**	**(1.02, 4.18)**	(0.85, 2.93)
Total physical activity	1.000	1.000	**1.002**	1.484
	(0.99, 1.01)	(0.99, 1.00)	**(1.00, 1.03)**	(0.98, 2.23)
Sedentary behaviour	0.610	0.746	**0.294**	**0.436**
	(0.32, 1.13)	(0.41, 1.33)	**(0.15, 0.54)**	**(0.25, 0.73)**
**Fitness**				
General Physical Condition	1.174	1.344	0.817	0.857
	(0.85, 1.62)	(0.98, 1.83)	(0.59, 1.12)	(0.65, 1.12)
Cardio-respiratory Fitness	1.238	1.320	0.845	0.899
	(0.92, 1.66)	(0.99, 1.75)	(0.63, 1.12)	(0.70, 1.15)
Muscular Strength	**1.914**	**1.777**	**1.391**	1.287
	**(1.36, 2.69)**	**(1.28, 2.46)**	**(1.02, 1.88)**	(0.99, 1.66)
Speed and Agility	1.301	**1.417**	1.136	1.081
	(0.94, 1.79)	**(1.04, 1.92)**	(0.83, 1.55)	(0.82, 1.41)
Flexibility	1.172	1.291	**1.331**	**1.346**
	(0.88, 1.55)	(0.98, 1.68)	**(1.00, 1.75)**	**(1.06, 1.70)**
General Health	1.312	**1.515**	0.996	1.223
	(0.92, 1.86)	**(1.08, 2.12)**	(0.71, 1.39)	(0.92, 1.62)

Analysis were adjusted for physical activity recommendations and Fitness self-reported (except in the analysis when that variable was the predictor variable). Notes: OR = Odd Ratio; 95% CI = 95% Confidence Intervals; Ref = reference; PA = Physical Activity; MVPA = Moderate to Vigorous Physical Activity; bold = Significant association with $p < 0.05$; and * = Private commuting was established as reference.

Table S2. Associations between PA recommendations and fitness by gender.

	**PA Recommendations ***	
	**Men**	**Women**
	**OR (95% CI)**	**OR (95% CI)**
**Fitness**		
General physical condition	**2.540 ****	**2.103 ****
	**(2.06, 3.11)**	**(1.73, 2.54)**
Cardio-respiratory fitness	**1.996 ****	**1.820 ****
	**(1.67, 2.38)**	**(1.53, 2.15)**
Muscular strength	**2.230 ****	**1.675 ****
	**(1.82, 2.72)**	**(1.40, 2.00)**
Speed and agility	**2.030 ****	**1.416 ****
	**(1.68, 2.44)**	**(1.18, 1.69)**
Flexibility	**1.460 ****	**1.385 ****
	**(1.24, 1.71)**	**(1.18, 1.62)**
General health	**2.020 ****	**1.495 ****
	**(1.65, 2.46)**	**(1.23, 1.80)**

Notes: OR = Odd Ratio; 95% CI = 95% Confidence Intervals; PA = Physical Activity; MVPA = Moderate to Vigorous Physical Activity; bold = Significant association with $p < 0.05$;** = $p < 0.001$; * = not meeting MVPA was established as reference.
